# Sodium Tanshinone IIA Sulfonate Prevents Angiotensin II-Induced Differentiation of Human Atrial Fibroblasts into Myofibroblasts

**DOI:** 10.1155/2018/6712585

**Published:** 2018-07-24

**Authors:** Tangting Chen, Miaoling Li, Xuehui Fan, Jun Cheng, Liqun Wang

**Affiliations:** ^1^Key Laboratory of Ministry of Education for Medical Electrophysiology and the Institute of Cardiovascular Research, Southwest Medical University, 319 Zhongshan Road, Luzhou, Sichuan 646000, China; ^2^Drug Discovery Research Center, Southwest Medical University, 319 Zhongshan Road, Luzhou, Sichuan 646000, China

## Abstract

Differentiation of atrial fibroblasts into myofibroblasts plays a critical role in atrial fibrosis. Sodium tanshinone IIA sulfonate (DS-201), a water-soluble derivative of tanshinone IIA, has been shown to have potent antifibrotic properties. However, the protective effects of DS-201 on angiotensin II- (Ang II-) induced differentiation of atrial fibroblasts into myofibroblasts remain to be elucidated. In this study, human atrial fibroblasts were stimulated with Ang II in the presence or absence of DS-201. Then, *α*-smooth muscle actin (*α*-SMA), collagen I, and collagen III expression and reactive oxygen species (ROS) generation were measured. The expression of transforming growth factor-*β*1 (TGF-*β*1) and the downstream signaling of TGF-*β*1, such as phosphorylation of Smad2/3, were also determined. The results demonstrated that DS-201 significantly prevented Ang II-induced human atrial fibroblast migration and decreased Ang II-induced *α*-SMA, collagen I, and collagen III expression. Furthermore, increased production of ROS and expression of TGF-*β*1 stimulated by Ang II were also significantly inhibited by DS-201. Consistent with these results, DS-201 significantly inhibited Ang II-evoked Smad2/3 phosphorylation and periostin expression. These results and the experiments involving N-acetyl cysteine (antioxidant) and an anti-TGF-*β*1 antibody suggest that DS-201 prevent Ang II-induced differentiation of atrial fibroblasts to myofibroblasts, at least in part, through suppressing oxidative stress and inhibiting the activation of TGF-*β*1 signaling pathway. All of these data indicate the potential utility of DS-201 for the treatment of cardiac fibrosis.

## 1. Introduction

Atrial fibrillation (AF), one of the most common arrhythmias, has become a serious epidemic across the world. Although atrial pathophysiology has been extensively studied, there are limited available therapies for patients with AF [1]. It is well known that atrial fibrosis which leads to atrial structural remodeling plays a pivotal role in the development and maintenance of AF [2, 3]. Inhibition of atrial fibrosis might be a plausible approach for AF prevention and therapy.

It has been demonstrated that atrial fibrosis is generally originated from nonmyocyte growth and extracellular matrix (ECM) protein deposition [4–6]. As the main nonmyocytes, fibroblasts undergo transdifferentiation into myofibroblasts, characterized by expression of contractile proteins, such as *α*-smooth muscle actin (*α*-SMA), and production of large amount of ECM components, such as collagen I and III, which are critically involved in atrial fibrosis [7, 8]. In addition, the development of atrial fibrosis and the myofibroblast differentiation have been shown to be highly regulated by angiotensin II (Ang II) and downstream activation of signaling pathway via soluble cytokines such as transforming growth factor-*β*1 (TGF-*β*1) [6, 9–11]. Therefore, the inhibition of Ang II-induced myofibroblast differentiation may be an important means for curing atrial fibrosis.

**Figure 1:**
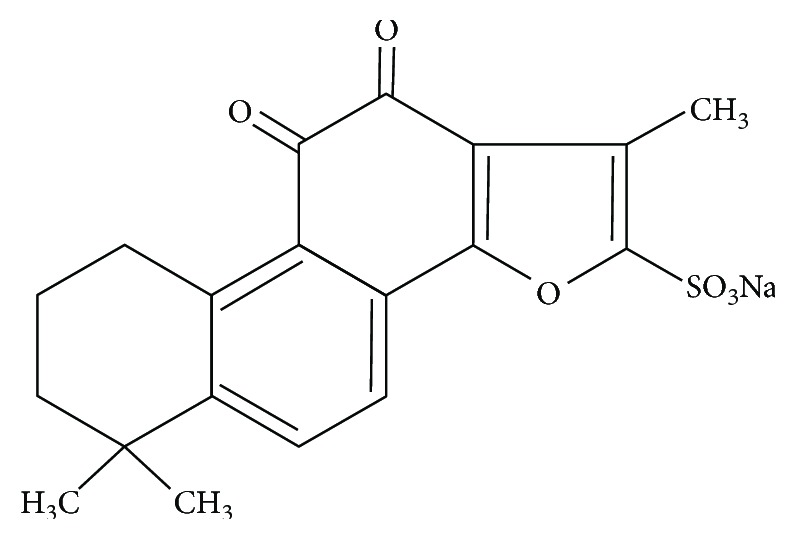
The molecular structure of DS-201.

Danshen, also known as *Salvia miltiorrhiza*, is a traditional Chinese herbal medicine that has been widely used for many years to treat various diseases including coronary artery disease, myocardial infarction, atherosclerosis, and cerebrovascular disorders [12, 13]. Sodium tanshinone IIA sulfonate (DS-201, molecular structure is shown in Figure 1) is a water-soluble derivative of tanshinone IIA which is one of the most pharmacologically active monomers extracted from danshen. It has been reported that DS-201 or tanshinone IIA can induce vasodilation [14–16], inhibit inflammatory response [17–19], and prevent atherosclerosis [20–23], cardiac injury [24, 25], and hypertrophy [26–28]. In addition, DS-201 or tanshinone IIA has been demonstrated to inhibit renal fibrosis [29, 30], bladder fibrosis [31], and pulmonary fibrosis [32, 33] and to prevent TGF-*β*-, radiation-, and hypertension-induced cardiac fibrosis [34–36]. Furthermore, tanshinone IIA has been clinically proven effective in treating the patients with liver fibrosis and severe pneumonia [37]. However, there are no studies that have examined the effects of DS-201 on Ang II-induced differentiation of atrial fibroblasts into myofibroblasts.

Thus, in the present study, we investigated the effects of DS-201 on Ang II-induced myofibroblast transdifferentiation, and our data indicate that DS-201 prevents Ang II-induced myofibroblast transdifferentiation via suppressing oxidative stress and TGF-*β*1 signaling pathway in human atrial fibroblasts.

## 2. Materials and Methods

### 2.1. Chemicals and Reagents

DS-201 (purity ≥ 98%) was obtained from the National Institutes for Food and Drug Control (Beijing, China). Ang II was purchased from Sigma (St Louis, MO, USA). Human adult atrial fibroblasts and fibroblast medium-2 were purchased from ScienCell (Carlsbad, CA, USA). Antibodies to *α*-SMA, phosphorylated Smad2/3, and total Smad2/3 were purchased from Santa Cruz Biotechnology (Santa Cruz, CA, USA). Antibodies to TGF-*β*1 and GAPDH were obtained from Cell Signaling Technology (Beverly, MA, USA). Antibodies to collagen I, collagen III, and periostin were obtained from Abcam (Cambridge, MA, USA). Cell Counting Kit 8 (CCK8) solution, 2′,7′-dichlorofluorescein diacetate (DCFH-DA), total superoxide dismutase (SOD) assay kits, catalase (CAT) assay kits, and n-acetyl cysteine (NAC) were purchased from Beyotime (Shanghai, China).

### 2.2. Cell Culture

Human adult atrial fibroblasts were cultured in fibroblast medium-2, and cells used were passaged 3–7 times.

### 2.3. Cell Viability Assays

Human atrial fibroblasts (1 × 10^4^/well) were seeded on 96-well plates and grown to confluence. Then, cells were starved of serum for 12 h, followed by stimulation with DS-201 (0, 5, 25, 50, 100, and 200 *μ*M) for 24 h. Cell viability was measured with CCK8 solution at 450 nm using a Spectra Max M5 microplate reader (Molecular Devices, Sunnyvale, CA, USA).

### 2.4. Cell Proliferation Assays

Human atrial fibroblasts (1 × 10^4^/well) were seeded on 96-well plates and grown to 50% confluence. Then, cells were starved of serum for 12 h, followed by stimulation with Ang II in the absence or in the presence of DS-201 (5, 25, 50, and 100 *μ*M) for 24 h. The cell proliferation was measured by adding 10 *μ*L CCK8 solutions into the wells and following 4 h incubation at 37°C. Then, the OD values of each well were measured by a Spectra Max M5 microplate reader at the absorbance of 450 nm.

### 2.5. Immunoblotting

Human atrial fibroblasts (3 × 10^5^/well) were plated on 3.5 cm wells, grown to confluence, and starved of serum for 12 h. Then, cells were exposed to Ang II (0.5 *μ*M) without or with DS-201 (5, 25, 50, and 100 *μ*M) for 24 h. In some experiments, fibroblasts were pretreated with NAC (10 mM) or an anti-TGF-*β*1 antibody (2 *μ*g/mL), followed by stimulation with Ang II (0.5 *μ*M) for 24 h. Then, cells were harvested and lysed in ice-cold RIPA lysis buffer (Beyotime) supplemented with protease and phosphatase inhibitors. The protein samples were separated with sodium dodecyl sulfate-polyacrylamide gel electrophoresis and transferred onto polyvinylidene fluoride membranes (Bio-Rad Laboratories, Hercules, CA, USA). The membranes were blocked with 5% nonfat dry milk solution for 1 h at room temperature. The blocked membranes were probed with antibodies against *α*-SMA (1 : 200), collagen I (1 : 1000), collagen III (1 : 1000), TGF-*β*1 (1 : 1000), phosphorylated Smad2/3 (1 : 200), total Smad2/3 (1 : 200), periostin (1 : 1000), and GAPDH (1 : 1000) overnight at 4°C. After washing with phosphate-buffered saline containing 0.05% Tween 20, the membranes were incubated with a horseradish peroxidase-conjugated secondary antibody (Santa Cruz Biotechnology) specific to the primary antibody. Then, the membranes were treated with enhanced chemiluminescence reagents (Merck Millipore, Watford, UK), and protein signal was imaged using ChemiDoc XRS (Bio-Rad Laboratories). ImageJ was used to measure the density of bands.

### 2.6. Cell Migration Assays

Cell migration was measured using transwell migration chambers with an 8.0 *μ*m-sized porous membrane (Corning Costar, Corning, NY, USA). Human atrial fibroblasts (3 × 10^4^) were added to the upper chambers and exposed to Ang II (0.5 *μ*M) without or with DS-201 (5, 25, 50, and 100 *μ*M) for 24 h. Then, the cells remaining in the upper chamber were removed using a cotton swab, and the membranes were fixed and then stained with 0.5% crystal violet. The cells that had migrated to the lower chamber were counted.

### 2.7. Measurement of Intracellular Reactive Oxygen Species (ROS)

Human atrial fibroblasts were exposed to Ang II (0.5 *μ*M) without or with DS-201 (5, 25, 50, and 100 *μ*M) for 1 h. Then, cells were washed with fibroblast medium-2 and subsequently incubated with DCFH-DA (10 *μ*M) for 30 min at 37°C. After incubation, the fluorescence intensity of the cells was determined using a Spectra Max M5 microplate reader (Molecular Devices, Sunnyvale, CA, USA) (488/525 nm), and the fluorescence images were also captured with an EVOS inverted microscope (AMG, Mill Creek, WA, USA).

### 2.8. Measurement of Intracellular SOD and CAT Level

Human atrial fibroblasts were exposed to Ang II (0.5 *μ*M) without or with DS-201 (5, 25, 50, and 100 *μ*M) for 1 h. Cell lysates were prepared, and the protein concentrations were determined using a BCA protein assay. Then, the level of SOD and CAT was measured by the respective kits according to the manufacturer's instructions.

### 2.9. Data Analysis

All data were expressed as mean ± standard deviation (SD) of the mean. Results were analyzed by one-way analysis of variance (ANOVA) followed by post hoc comparison. *P* < 0.05 was considered to be significantly different.

## 3. Results

### 3.1. Effects of DS-201 on Cell Viability

**Figure 2:**
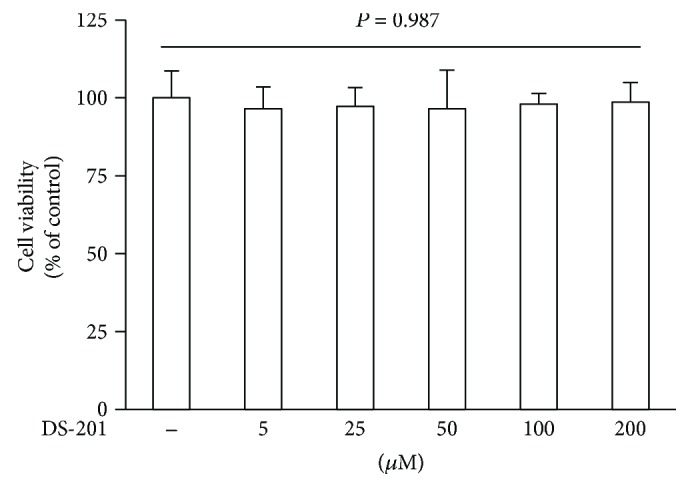
Effects of DS-201 on cell viability. Human atrial fibroblasts were exposed to DS-201 (0, 5, 25, 50,100, and 200 *μ*M) for 24 h. Cell viability was measured using the CCK8 assay. Data shown are mean ± SD of 4 independent experiments, presented as % of the control value (first bar).

To identify whether DS-201 treatment induced cell death, the effects of DS-201 on fibroblast viability were detected. Human atrial fibroblasts were stimulated with DS-201 (0, 5, 25, 50, 100, and 200 *μ*M) for 24 h, and cell viability assay was performed with a CCK8 solution. The results showed no significant differences among the groups (Figure 2), indicating that at the maximum concentration of 200 *μ*M, DS-201 did not affect fibroblast viability. Therefore, the highest concentration of DS-201 we used was 100 *μ*M in the follow-up experiments.

### 3.2. DS-201 Prevents Ang II-Induced Fibrotic Response in Human Atrial Fibroblasts

**Figure 3:**
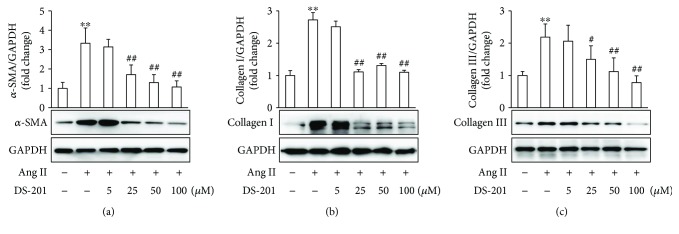
DS-201 prevents Ang II-induced fibrotic response in atrial fibroblasts. Atrial fibroblasts were exposed to Ang II (0.5 *μ*M) with or without DS-201 (0, 5, 25, 50, and 100 *μ*M) for 24 h. (a) Expression of *α*-SMA was analyzed by western blotting, and representative images of 3 independent experiments are shown. The ratio of *α*-SMA normalized to GAPDH was calculated. (b) Expression of collagen I was analyzed by western blotting, and representative images of 3 independent experiments are shown. The ratio of collagen I normalized to GAPDH was calculated. (c) Expression of collagen III was also analyzed by western blotting, and representative images of 3 independent experiments are shown. The ratio of collagen III normalized to GAPDH was calculated. All data shown are mean values ± SD and are expressed as fold changes. ^∗∗^ *P* < 0.01 versus control (first bar); ^#^ *P* < 0.05 versus Ang II; ^##^ *P* < 0.01 versus Ang II.

Fibrotic response in atrial fibroblasts, involving fibroblast differentiation to myofibroblast, fibroblast-derived ECM protein deposition, fibroblast migration, and proliferation, plays an import role in atrial fibrosis. To explore the effects of DS-201 on Ang II-induced fibrotic response, myofibroblast differentiation and ECM protein deposition were firstly investigated. Atrial fibroblasts were exposed to Ang II (0.5 *μ*M) in the absence or presence of DS-201 (0, 5, 25, 50, and 100 *μ*M) for 24 h. Then, cell lysates were prepared, and the expression of *α*-SMA, collagen I, and collagen III was determined with western blotting. The data showed that *α*-SMA, collagen I, and collagen III expression significantly increased with Ang II stimulation but decreased by DS-201 treatment (Figure 3). We also examined the effects of DS-201 alone on *α*-SMA, collagen I, and collagen III expression, and the results showed no significant differences among the groups (Supplementary Figure 1 and Supplementary Figure 2). The results indicate that DS-201 prevents Ang II-induced myofibroblast differentiation and ECM protein expression.

**Figure 4:**
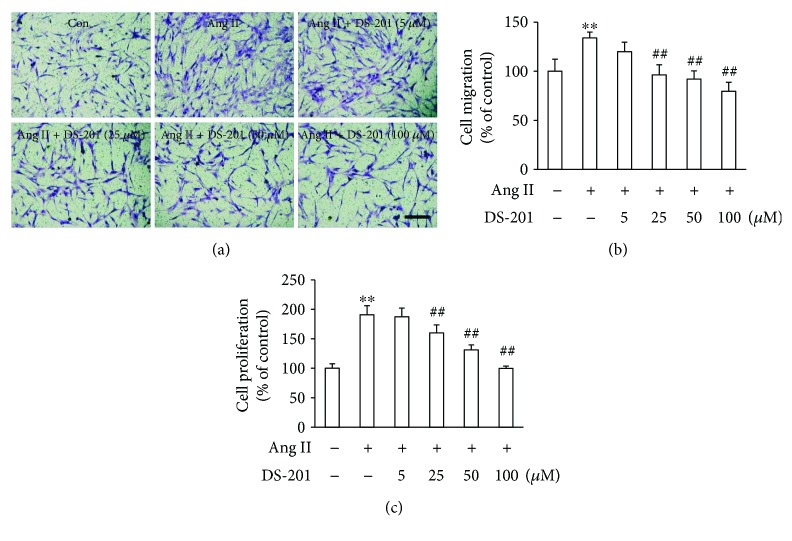
DS-201 prevents Ang II-induced cell migration and cell proliferation. (a) Atrial fibroblasts were added to upper chambers containing porous filters and then treated with Ang II (0.5 *μ*M) with or without DS-201 (0, 5, 25, 50, and 100 *μ*M). After 24 h, the cells were fixed and were stained with crystal violet. The cells that had migrated into the lower chambers were counted. Representative images of cell migration are shown (scale bar, 200 *μ*m). (b) Quantitative assessment of 3 independent cell migration experiments was performed. (c) Atrial fibroblasts were stimulated with Ang II in the absence or in the presence of DS-201 (5, 25, 50, and 100 *μ*M) for 24 h. Then, cell proliferation was measured by CCK8 assay. All data shown are mean values ± SD and are expressed as % of the control value (first bar). ^∗∗^ *P* < 0.01 versus control (first bar); ^##^ *P* < 0.01 versus Ang II.

The migration of fibroblast was also detected using a transwell chamber migration assay. Human atrial fibroblasts were added to the upper chamber containing porous filters and treated with Ang II (0.5 *μ*M) with or without DS-201 (0, 5, 25, 50, and 100 *μ*M). After 24 h, the cells that had migrated through the membrane were counted. The results showed that Ang II significantly increased the migrated cells compared with that in the vehicle control group, and DS-201 treatment significantly inhibited Ang II-induced fibroblast migration (Figures 4(a) and 4(b)). The effects of DS-201 on Ang II-induced fibroblast proliferation were also determined. The data showed that the cell proliferation significantly increased by Ang II stimulation but decreased with DS-201 treatment (Figure 4(c)). As a whole, all of these results suggest that DS-201 prevent Ang II-induced fibrotic response in human atrial fibroblasts.

### 3.3. DS-201 Prevents Ang II-Induced Oxidative Stress

**Figure 5:**
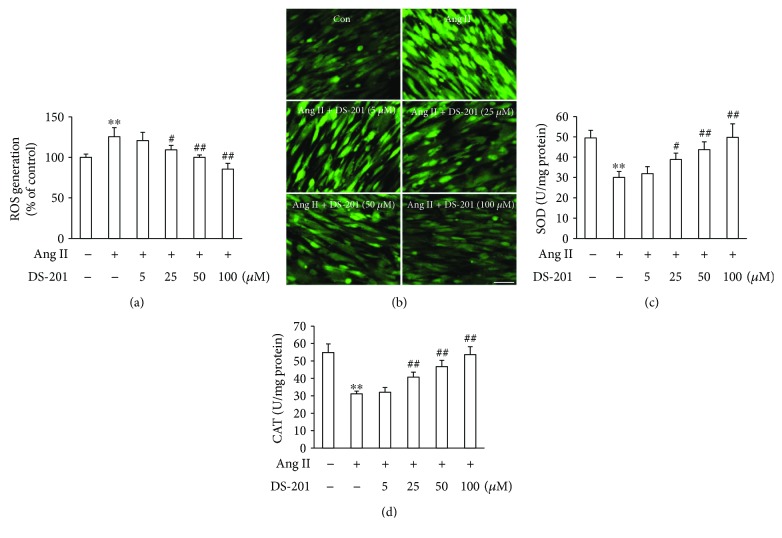
DS-201 inhibits Ang II-induced oxidative stress. Atrial fibroblasts were exposed to Ang II (0.5 *μ*M) with or without DS-201 (0, 5, 25, 50, and 100 *μ*M) for 1 h. (a) Cells were stained with DCFH-DA and the fluorescence intensity of DCF was measured at 488/525 nm using a microplate reader. (b) The DCF fluorescence was also monitored by fluorescence microscopy, and representative images of 3 independent experiments are shown (scale bar, 100 *μ*m). (c, d) The level of SOD and CAT was measured by the respective kits according to the manufacturer's instructions. All data shown are mean ± SD for 3 independent experiments and presented as % of the control value (first bar). ^∗∗^ *P* < 0.01 versus Con; ^#^ *P* < 0.05 versus Ang II; ^##^ *P* < 0.01 versus Ang II.

Since overproduction of ROS has been demonstrated to contribute to atrial fibrosis [38, 39], the effects of DS-201 on Ang II-induced ROS generation were detected. Atrial fibroblasts were stimulated with Ang II (0.5 *μ*M) in the absence or presence of DS-201 (0, 5, 25, 50, and 100 *μ*M) for 1 h. Then, cells were stained with DCFH-DA, and the fluorescence intensity of DCF was measured at 488/525 nm using a microplate reader. The results showed that intracellular ROS significantly increased in Ang II-treated fibroblast but decreased by DS-201 treatment (Figure 5(a)). Similar results were also shown in fluorescence images (Figure 5(b)). The activity of the antioxidant enzymes, such as SOD and CAT, was also measured. The data showed that the SOD and CAT levels significantly decreased by Ang II stimulation but restored with DS-201 treatment (Figures 5(c) and 5(d)). We also examined the effects of DS-201 alone on ROS generation, and the results showed no significant differences among the groups (Supplementary Figure 3). These data indicate that DS-201 prevents Ang II-induced oxidative stress.

**Figure 6:**
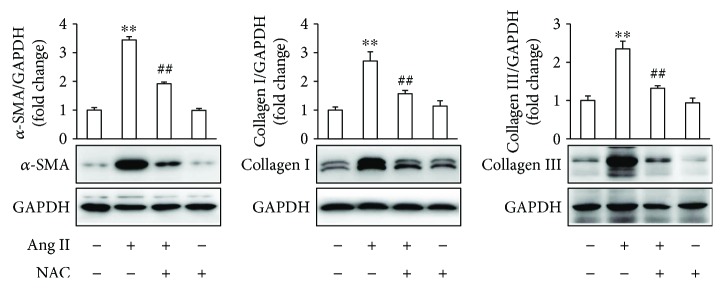
NAC inhibits Ang II-induced fibrotic response in atrial fibroblasts. Atrial fibroblasts were pretreated with NAC (10 mM) for 1 h, followed by stimulation with Ang II (0.5 *μ*M) for 24 h. Expression of *α*-SMA, collagen I, and collagen III was analyzed by western blotting, and representative images of 3 independent experiments are shown. The ratio of *α*-SMA, collagen I, and collagen III normalized to GAPDH was calculated. All data shown are mean values ± SD and are expressed as fold changes. ^∗∗^ *P* < 0.01 versus control (first bar); ^##^ *P* < 0.01 versus Ang II.

To further determine the effects of antioxidant on Ang II-induced fibrotic response in atrial fibroblasts, the cells were pretreated with NAC for 1 h, followed by stimulation with Ang II for 24 h. The results demonstrated that NAC significantly inhibited Ang II-induced *α*-SMA expression and ECM deposition (Figure 6). As a whole, these results indicate that DS-201 prevents Ang II-induced differentiation of atrial fibroblasts to myofibroblasts through inhibiting oxidative stress.

### 3.4. DS-201 Prevents Ang II-Induced TGF-*β*1 Activation

**Figure 7:**
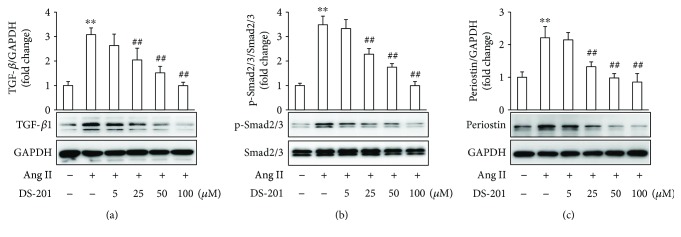
DS-201 prevents Ang II-induced TGF-*β*1 activation in human atrial fibroblasts. Atrial fibroblasts were exposed to Ang II (0.5 *μ*M) with or without DS-201 (0, 5, 25, 50, and 100 *μ*M) for 24 h. (a) Expression of TGF-*β*1 was analyzed by western blotting, and representative images of 3 independent experiments are shown. The ratio of TGF-*β*1 normalized to GAPDH was calculated. (b) Phosphorylation (p) of Smad2/3 was analyzed by western blotting. Representative images of 3 independent experiments and densitometric analysis of phosphorylated Smad2/3 normalized to total Smad2/3 are shown. (c) Expression of periostin was analyzed by western blotting, and representative images of 3 independent experiments are shown. The ration of periostin normalized to GAPDH was calculated. ^∗∗^ *P* < 0.01 versus control (first bar); ^##^ *P* < 0.01 versus Ang II.

Previous studies have demonstrated that TGF-*β*1 plays a key role in atrial fibrosis [11, 39]. Specially, it was shown that in the absence of TGF-*β*1, Ang II was not able to induce cardiac hypertrophy and fibrosis in vivo [40]. Therefore, the effects of DS-201 on Ang II-induced TGF-*β*1 activation were investigated. Atrial fibroblasts were exposed to Ang II (0.5 *μ*M) with or without DS-201 (0, 5, 25, 50, and 100 *μ*M) for 24 h. Cell lysates were prepared, and the protein was analyzed using western blotting. The results demonstrated that the expression of TGF-*β*1 significantly increased with Ang II stimulation but decreased in response to DS-201 treatment (Figure 7(a)). We also found that DS-201 alone had no effects on TGF-*β*1 expression (Supplementary Figure 4).

The phosphorylation of Smad2/3, the downstream signaling of TGF-*β*1, was also determined. The data showed that DS-201 significantly inhibited Ang II-evoked Smad2/3 phosphorylation (Figure 7(b)). Furthermore, periostin is well known to be a TGF-*β*1-inducible matrix protein. We then investigated whether DS-201 affects periostin expression. The results showed that the expression of periostin significantly increased in response to Ang II, whereas DS-201 decreased its expression (Figure 7(c)). Taken together, these results suggest that DS-201 prevents Ang II-induced TGF-*β*1 activation.

**Figure 8:**
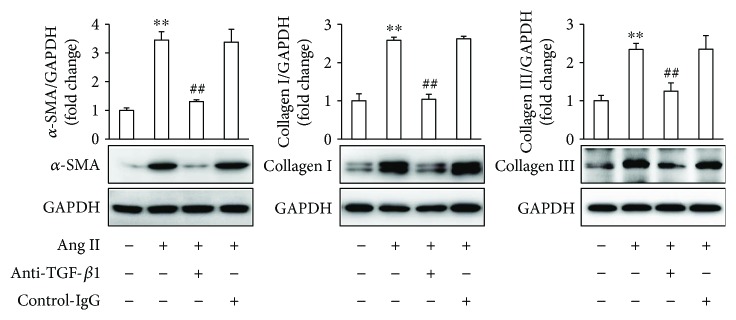
The blockade of TGF-*β*1 inhibits Ang II-induced fibrotic response in atrial fibroblasts. Atrial fibroblasts were pretreated with an anti-TGF-*β*1 antibody (2 *μ*g/mL) for 1 h, followed by stimulation with Ang II (0.5 *μ*M) for 24 h. Expression of *α*-SMA, collagen I, and collagen III was analyzed by western blotting, and representative images of 3 independent experiments are shown. The ratio of *α*-SMA, collagen I, and collagen III normalized to GAPDH was calculated. All data shown are mean values ± SD and are expressed as fold changes. ^∗∗^ *P* < 0.01 versus control (first bar); ^##^ *P* < 0.01 versus Ang II.

To further identify the effects of blockade of TGF-*β*1 on Ang II-induced fibrotic response in atrial fibroblasts, the cells were pretreated with an anti-TGF-*β*1 antibody for 1 h, followed by stimulation with Ang II for 24 h. The results demonstrated that the anti-TGF-*β*1 antibody significantly inhibited Ang II-induced *α*-SMA expression and ECM deposition (Figure 8). As a whole, these results indicate that DS-201 prevents Ang II-induced differentiation of atrial fibroblasts to myofibroblasts through inhibiting the activation of TGF-*β*1 signaling pathway.

## 4. Discussion

It is well known that Ang II, which has been reported to be activated in various cardiovascular diseases, such as myocardial infarction and hypertension [41, 42], plays a major role in atrial fibrosis by promoting differentiation of atrial fibroblasts into myofibroblasts [43]. Therefore, the identification of new preventive and therapeutic approaches targeting Ang II-induced myofibroblast differentiation has great clinical implications. In the present study, we evaluated the effects of DS-201 on Ang II-induced atrial fibrosis, and the results demonstrated that DS-201 prevented Ang II-induced myofibroblast differentiation via inhibiting oxidative stress and downregulating TGF-*β*1 signaling pathway in human atrial fibroblasts.

The differentiation of fibroblasts into myofibroblasts is characterized by *α*-SMA expression and ECM protein deposition. Previous reports [44–46] and our data in this study demonstrated that Ang II significantly increased fibroblast migration, *α*-SMA expression, and collagen production. However, the treatment with DS201 prevented Ang II-induced fibrotic response in atrial fibroblasts in a dose-dependent manner. These results were consistent with previous studies which showed that DS-201 inhibited TGF-*β*-, radiation-, and hypertension-induced cardiac fibrosis [34–36].

Growing evidence has highlighted oxidative stress as an important mechanism in pathologic cardiac remodeling [38, 39]. Previous reports [44, 47] and our results demonstrated that Ang II significantly increased intracellular ROS generation in fibroblasts. The present results clearly showed that DS-201 significantly inhibited Ang II-induced ROS generation and increased the activation of enzymes which can scavenge ROS such as SOD and CAT in atrial fibroblasts, which was in line with these reported by other investigators [48, 49]. Although the mechanisms by which ROS mediates the differentiation of atrial fibroblasts into myofibroblasts remain unclear, our experiments involving NAC suggest that the inhibition of oxidative stress significantly prevents Ang II-induced fibrotic response in atrial fibroblasts. These results were consistent with previous studies which have shown that ROS is necessary for Ang II- or TGF-*β*1-induced *α*-SMA expression and myofibroblast differentiation [44, 50]. Thus, the present findings strongly suggest that DS-201 prevents Ang II-induced differentiation of atrial fibroblasts to myofibroblasts by blocking oxidative stress.

TGF-*β*1 plays an important role in the pathogenesis of cardiac remodeling and fibrosis. In genetic modification studies, TGF-*β*1 overexpression in the mouse heart was associated with fibrosis [51, 52]. In addition, extensive evidence has suggested a direct link between the renin-angiotensin system and TGF-*β*1, indicating that TGF-*β*1 acts downstream of Ang II [53]. Consistent with previous studies, our data also showed that Ang II significantly increased TGF-*β*1 expression and Smad2/3 phosphorylation, whereas treatment with DS-201 dose-dependently inhibited increased the expression of TGF-*β*1 and activation of Smad2/3. Periostin, a TGF-*β*-inducible matrix protein, has been demonstrated to contribute to fibrosis by regulating ECM molecules such as collagen and fibronectin [54]. Periostin knockout resulted in reduced fibrosis and hypertrophy after pressure overload, whereas periostin-overexpressing transgenic mice develop spontaneous hypertrophy with aging [55, 56]. Consistent with these reports, our results showed that periostin expression was massively increased by Ang II stimulation, but its expression was decreased with a concomitant reduction in fibrosis after DS-201 treatment.

Although few previous studies focused the effects of DS-201 on Ang II-induced TGF-*β*1 signaling pathway activation, previous reports have indicated that tanshinone IIA attenuated TGF-*β*1 expression in pulmonary fibrosis [32] and in chronic kidney disease [57], which were partly in agreement with our results. Furthermore, our experiments involving an anti-TGF-*β*1 antibody indicate that the blockade of TGF-*β*1 signaling pathway significantly inhibits Ang II-induced fibrotic response. Taken together, the present results suggest that DS-201 prevents Ang II-induced differentiation of atrial fibroblasts to myofibroblasts through downregulating TGF-*β*1 signaling pathway.

Based on our results in the present study and those of published reports, it is conceivable that DS-201 has implications for atrial fibrosis prevention and therapy. However, the underlying mechanisms, especially, the target of DS-201, have not been fully elucidated. Therefore, further investigations are needed to better explore the therapeutic potential of DS-201.

## 5. Conclusions

In summary, we have described a protective role of DS-201 in Ang II-induced differentiation of atrial fibroblasts to myofibroblasts and the molecular mechanism involved in the present study. Our data demonstrated that DS-201 prevented Ang II-induced atrial fibrosis through inhibiting oxidative stress and suppressing TGF-*β*1 signaling pathway. These novel findings indicate the potential application of DS-201 for the prevention and treatment of fibrosis disease in the clinical practice.

## Data Availability

The data used to support the findings of this study are available from the corresponding author upon request.

## Supplementary Materials

Supplementary Materials This supplementary material file contains 4 figures. These results show the effects of DS-201 on *α*-SMA, collagen I, collagen III, and TGF-*β*1 expression and ROS generation. Supplementary Figure 1: the effects of DS-201 on *α*-SMA expression. Atrial fibroblasts were exposed to DS-201 (0, 5, 25, 50, and 100 *μ*M) for 24 h, and then the expression of *α*-SMA was analyzed by western blotting. Supplementary Figure 2: the effects of DS-201 on collagen I and collagen III expression. Atrial fibroblasts were exposed to DS-201 (0, 5, 25, 50, and 100 *μ*M) for 24 h, and then the expression of collagen I and collagen III was analyzed by western blotting. Supplementary Figure 3: the effects of DS-201 on ROS generation. Atrial fibroblasts were exposed to DS-201 (0, 5, 25, 50, and 100 *μ*M) for 1 h, and then the production of ROS was measured by DCFH-DA staining. Supplementary Figure 4: the effects of DS-201 on TGF-*β*1 expression. Atrial fibroblasts were exposed to DS-201 (0, 5, 25, 50, and 100 *μ*M) for 24 h, and then the expression of TGF-*β*1 was analyzed by western blotting.
