# Supplementary material for: Sodium Tanshinone IIA Sulfonate Prevents Angiotensin II-Induced Differentiation of Human Atrial Fibroblasts into Myofibroblasts
Source: Oxid Med Cell Longev. 2018 Jul 24;2018:6712585. doi: 10.1155/2018/6712585 (PMC6081515; doi:10.1155/2018/6712585)
Supplement: Supplementary Materials — This supplementary material file contains 4 figures. These results show the effects of DS-201 on α-SMA, collagen I, collagen III, and TGF-β1 expression and ROS generation. Supplementary Figure 1: the effects of DS-201 on α-SMA expression. Atrial fibroblasts were exposed to DS-201 (0, 5, 25, 50, and 100 μM) for 24 h, and then the expression of α-SMA was analyzed by western blotting. Supplementary Figure 2: the effects of DS-201 on collagen I and collagen III expression. Atrial fibroblasts were exposed to DS-201 (0, 5, 25, 50, and 100 μM) for 24 h, and then the expression of collagen I and collagen III was analyzed by western blotting. Supplementary Figure 3: the effects of DS-201 on ROS generation. Atrial fibroblasts were exposed to DS-201 (0, 5, 25, 50, and 100 μM) for 1 h, and then the production of ROS was measured by DCFH-DA staining. Supplementary Figure 4: the effects of DS-201 on TGF-β1 expression. Atrial fibroblasts were exposed to DS-201 (0, 5, 25, 50, and 100 μM) for 24 h, and then the expression of TGF-β1 was analyzed by western blotting. [file 6712585.f1.pdf]

**Supplementary Figures:**

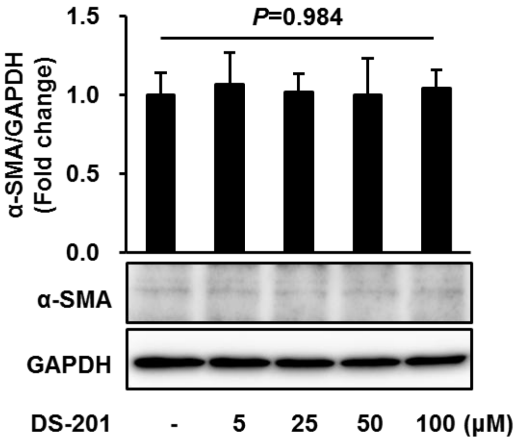


**Supplementary Figure 1:** The effects of DS-201 on α-SMA expression. Atrial fibroblasts were exposed to DS-201 (0, 5, 25, 50 and 100 μM) for 24 h. Expression of α-SMA was analyzed by western blotting and representative images of 3 independent experiments are shown. The ratio of α-SMA normalized to GAPDH was calculated. Data shown are mean values ± SD and are expressed as fold changes.

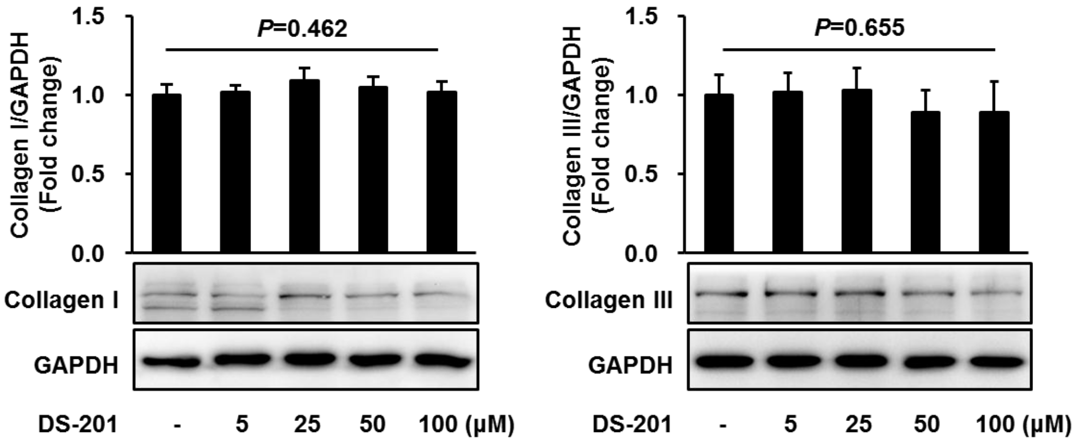


**Supplementary Figure 2:** The effects of DS-201 on collagen I and collagen III expression. Atrial fibroblasts were exposed to DS-201 (0, 5, 25, 50 and 100 μM) for 24 h. Expression of collagen I and collagen III was analyzed by western blotting and representative images of 3 independent experiments are shown. The ratio of collagen I and collagen III normalized to GAPDH was calculated. Data shown are mean values ± SD and are expressed as fold changes.

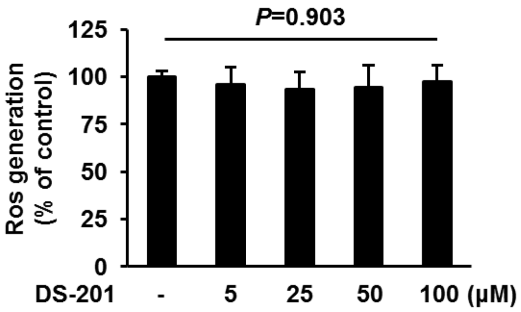


**Supplementary Figure 3:** The effects of DS-201 on ROS generation. Atrial fibroblasts were exposed DS-201 (0, 5, 25, 50 and 100 μM) for 1 h. Then cells were stained with DCFH-DA. The fluorescence intensity of DCF was measured at 488/525 nm using a microplate reader. Data shown are mean ± SD for 3 independent experiments and presented as % of the control value (first bar).

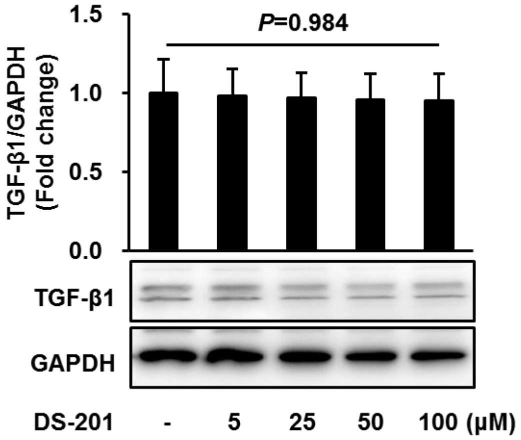


**Supplementary Figure 4:** The effects of DS-201 on TGF-β1 expression. Atrial fibroblasts were exposed to DS-201 (0, 5, 25, 50 and 100 μM) for 24 h. Expression of TGF-β1 was analyzed by western blotting and representative images of 3 independent experiments are shown. The ratio of TGF-β1 normalized to GAPDH was calculated. Data shown are mean values ± SD and are expressed as fold changes.
